# One-year outcome in patients with idiopathic normal-pressure hydrocephalus treated with a lumbo-peritoneal shunt (SINPHONI-2), compared to ventriculo-peritoneal shunt (SINPHONI) used as a historical control

**DOI:** 10.1186/2045-8118-12-S1-O46

**Published:** 2015-09-18

**Authors:** Masakazu Miyajima, Hiroaki Kazui, Etsuro Mori, Masatsune Ishikawa

**Affiliations:** 1 Department of Neurosurgery, Juntendo University Graduate School of Medicine, Japan; 2 Department of Psychiatry, Osaka University Graduate School of Medicine, Japan; 3 Department of Behavioral Neurology and Cognitive Neuroscience, Tohoku University Graduate School of Medicine, Japan; 4 Normal-Pressure Hydrocephalus Centre, Otowa Hospital, Japan

## Introduction

Idiopathic normal pressure hydrocephalus (INPH) is treated with cerebrospinal fluid shunting, and ventriculo-peritoneal shunt (VPS) is the current standard treatment. The goal of this pooled analysis was to compare the efficacy and safety between VPS and lumbo-peritoneal shunt (LPS) in patients with INPH specified as disproportionately enlarged subarachnoid space hydrocephalus (DESH).

## Methods

We conducted a multicenter prospective 3-month randomized controlled trial, and then a 1-year extension study, where all subjects received an LPS with a programmable valve and were examined periodically for 1 year. Eighty-three patients with INPH (60 to 85 years old) presenting with ventriculomegaly, high-convexity and medial subarachnoid space tightness in magnetic resonance imaging were recruited from 20 neurological or neurosurgical centers in Japan between March 1, 2010 and October 19, 2011. The primary outcome was the modified Rankin scale (mRS) score 1 year after surgery, and secondary outcome included the NPH grading scale (NPHGS). The VPS SINPHONI study was used as a historical control.

## Results

The proportion of patients with a favorable outcome (i.e., improvement of at least one level in mRS) was 63% (95% CI: 51-73%), and was comparable to that with VPS implantation (69%, 95% CI: 59-78%). In NPHGS, the one-year improvement rate was 75% (95% CI: 64-84%) and was comparable to that of VPS (77%, 95% CI: 68-84%). Serious adverse events (SAEs) occurred in 19 patients (22%), 10 of which were related to surgery. SAEs were more common with LPS than with VPS (15%).

## Conclusion

Our results show that LPSs with programmable valves are effective for treating INPH as an alternative to VPSs.
